# Factors Associated with Smartphone Addiction Tendency in Korean Adolescents

**DOI:** 10.3390/ijerph182111668

**Published:** 2021-11-06

**Authors:** Ji-Hye Kim

**Affiliations:** College of Nursing, Woosuk University, Jeonju-si 55338, Korea; jihye2020@woosuk.ac.kr; Tel.: +82-63-290-1749

**Keywords:** adolescents, smartphone addiction, protective factor, social support, bullying

## Abstract

This study aims to identify the factors associated with smartphone addiction tendency in Korean adolescents. A convenience sample of 502 students from four middle schools participated in the survey for the assessment of demographic, personal factors (resilience and academic stress), environmental factors (parental support, teacher support, friend support, and bullying victimization), as well as smartphone addiction tendency. Smartphone addiction tendency was determined based on the Smartphone Addiction Proneness Scale for Youth (SAPS) criteria developed by Kim et al. The collected data were analyzed using descriptive statistics, chi-squared test, *t*-test, Pearson’s correlation coefficients, and hierarchical logistic regression. Among the adolescents, 17.9% were in the smartphone addiction tendency group. The factors associated with smartphone addiction tendency were subjective economic level, academic stress, parental support, and bullying victimization. Based on the findings of this study, it is considered necessary to approach smartphone addiction management considering personal factors as well as environmental factors.

## 1. Introduction

The National Information Society Agency (NIA) reported that 23.3% of smartphone users in 2020 suffered smartphone addiction in South Korea [1]. Specifically, adolescents showed the highest figure of 39.6% [1], which was higher than in other countries [2,3].

Smartphone addiction refers to a state in which users cannot control their smartphone use by themselves, and experience withdrawal and tolerance for smartphone use due to excessive smartphone use, which causes disturbances in daily life [4]. In recent studies, smartphone addiction is regarded as behavioral addiction and one of the problematic behaviors of adolescents [5,6,7]. Behavioral addiction refers to a continuous repetition of certain behaviors that cause physiological and psychological difficulties [6]. It can be characterized by loss of control, withdrawal, tolerance, mood modification, and conflict [6].

Smartphone addiction in adolescence can cause problems in various aspects. For example, excessive use of smartphones threatens physical health by causing sleep disturbance; reduced vision; pain in the cervical spine, wrists, and shoulders; and secondary problems due to lack of exercise [8,9]. It has negative effects on mental health such as depression, anxiety, increased aggression, and poor concentration [7,8,10]. In addition, social problems such as decreased academic performance, accidents, family conflicts, and increased exposure to pornography due to excessive use of smartphones are also emerging [11,12].

Adolescence is a critical period in establishing identity and preparing for a successful transition into adulthood [13]. In addition, adolescence is a period of high curiosity about new things and low self-control, which makes them vulnerable to smartphone addiction [5]. The psychological and social problems mentioned above caused by the overuse of smartphones not only affect adolescents’ identification, but also the overall life of adolescence and health and well-being of adulthood [13,14]. For example, depression and anxiety caused by overuse of smartphones are related to the well-being of adolescents, which may affect psychological problems later in adulthood [15]. Considering the developmental characteristics of adolescence, it is necessary to prevent and manage smartphone addiction.

According to prior studies, smartphone addiction in adolescents is related to personal factors such as stress, depression, anxiety, impulsivity, and personality [16,17,18]. Stress has been identified as a major factor in research on adolescents’ problematic behaviors [19]. In particular, academic stress is a major source of daily stress for Korean adolescents, which has a positive relationship with depression and anxiety [20]. Therefore, it is necessary to identify its relationship with smartphone addiction in Korean adolescents who suffer from academic stress, a major stress of adolescents. 

Adolescent behaviors are determined by their interactions with the systems surrounding them at various levels (family, school, community, etc.) [21]. As adolescents live in a limited environment such as family and school, the parents, teachers, and friends they build a relationship with serve as important social supports [22,23]. Once adolescents perceive the support of their parents, teachers, and friends, they feel psychological stability and improved self-control [24], which can affect the risk of smartphone addiction. Recently, peer victimization has been identified as a risk factor for smartphone addiction [25]. Among the types of school victimization, bullying victimization, in particular, is related to a greater risk of smartphone addiction as it is in relation to adolescents’ internalizing problems (e.g., depression, anxiety, and loneliness) and overuse of Social Media [26,27]. In order to understand the behavior of adolescents, it is essential to consider the adolescent’s environment, but few studies have examined the predictors of smartphone addiction in adolescents from a multidimensional perspective [21,22,23]. Hence, it is necessary to conduct a study on how the support from parents, teachers, and friends—the major environment surrounding adolescents—and bullying victimization that causes psychological issues, are associated with smartphone addiction.

In addition, studies on the protective factors of smartphone addiction are relatively lacking although various protective factors play an important role in problematic adolescent behaviors [19]. Protective factors directly influence adolescents to engage in desirable behaviors or serve as a buffer to reduce the impact of risk factors [28]. They were identified as protective factors for problematic behaviors or internet addiction in adolescents including resilience, self-control, social support, and peer relationships [28,29]. Resilience refers to “the ability to maintain adaptive functions in adversities or at-risk situations” and is a fundamental concept of the health and well-being of adolescents [30]. Resilience of adolescents plays an intermediary role between stressful situations and psychological well-being of adolescents while regulating the control of problematic behaviors [30]. It is empirically supported that such protective factors not only affect the educational aspects such as school adjustment and academic achievement but are also a major factor in mental health problems such as depression, anxiety, and suicidal thoughts [30,31]. Therefore, it is necessary to search for protective factors that can control negative effects of risk factors associated with smartphone addiction tendency and directly suppress such a tendency.

As smartphone addiction in adolescents has emerged as a social issue, there has been a surge in relevant research. However, most studies have concentrated primarily on the risk factors of individuals, leaving insufficient research on the environmental aspects and protective factors of adolescents. It is meaningful to identify the relationship between smartphone addiction tendency and academic stress; resilience; bullying victimization; and support from parents, teachers, and friends, to understand adolescent behaviors.

According to the social ecological model, health behaviors of human beings are influenced by intrapersonal, interpersonal, institutional, community, and political factors [32]. The strength of the social ecological model is that it enables a multidimensional and holistic approach to health behaviors, as it looks at health behaviors from a social context. Based on the social ecological model, this study aims to examine the relevant factors of smartphone addiction tendency among Korean adolescents by focusing on personal (intrapersonal) and environmental (interpersonal) factors. This study aimed to determine the prevalence of smartphone addiction tendency among adolescents in Korea (Aim 1) and identify risk factors and protective factors in terms of personal and environmental factors that are associated with adolescents’ smartphone addiction tendency (Aim 2). The findings can be used as foundational data for developing intervention strategies to prevent smartphone addiction in adolescents.

## 2. Materials and Methods

### 2.1. Study Design

This study is a descriptive and cross-sectional study investigating the factors associated with smartphone addiction tendency in Korean adolescents.

### 2.2. Study Participants and Data Collection

The population involved students attending four public middle schools located in Korean cities (Seoul, Busan, Cheongju, Gwangmyeong). For the study, two co-education schools, one boy-only middle school, and one girl-only middle school, were conveniently sampled. The participants of this study were adolescents aged ≥12 years and ≤18, who currently use smartphones and voluntarily consented to participate in the study. Adolescents who received counseling and treatment for psychiatric problems in the past year were excluded from this study. The sample size of this study was calculated under the conditions of the medium-effect size of logistic regression analysis (odds ratio (OR) = 1.5, H0 = 0.2, X parm μ = 2, X parm σ = 1), significance level of 0.05, power of 0.90, and two-tailed test using G*Power 3.1 software [33]. The minimum sample size was 295. In this study, the questionnaire was distributed to 700 people considering the potential withdrawals; 553 questionnaires of them were collected. Thirty-three questionnaires that responded in a certain pattern (answered all questions with the same number) and 18 questionnaires with missing data were excluded. A total of 502 questionnaires were finally analyzed. The mean age of the participants was 13.0 ± 0.80 years (range: 12~17 years), and 275 participants were females (54.8%).

This study was approved by the Institutional Review Board of H-University to which the researcher was affiliated (No. HYI-18-140-1). Data were collected from 26 November to 20 December 2018. The participants were informed of the study’s purpose, procedure, voluntary participation, anonymity, confidentiality, and the freedom to withdraw consent without any disadvantages. Subjects who agreed to participate in the study were provided with a questionnaire, a study description, and caregiver/participant consent form. If the caregiver and participant agreed to participate in the study, they were asked to sign the consent form and complete the questionnaire. Completed questionnaires were inserted in a sealed envelope and were collected by the researcher.

### 2.3. Measurements

#### 2.3.1. Sociodemographic Characteristics

As sociodemographic characteristics: sex, age, type of school system, structure of family, parental working status, subjective economic level, and subjective health status were included. The type of school system was classified into “co-education, boys, and girls”. The structure of family was classified into “Living with both parents” and “Not living with both parents”, and the working status of parents was divided into “Both parents working” and “Single parent working/Other”. The subjective economic level was classified into “high, medium, low”. The subjective health status was classified into “healthy and unhealthy”.

#### 2.3.2. Personal Factors

Personal factors included resilience and academic stress. Resilience was measured using the Korean version of RS-14 [34]. The Korean version of RS-14 is an adaptation of Wagnild’s 14-Item Resilience Scale (RS-14) to verify validity and reliability [35]. The Korean version of RS-14 consists of one factor and 14 items, and each item was rated on a 7-point Likert scale (1 = strongly disagree, 7 = strongly agree). The higher the score, the higher the resiliency. Cronbach’s α was 0.93 in a previous study [35] and 0.92 in this study.

Academic stress was measured using the Academic Stress Scale developed by Park and Kim [36]. It measures the degree of academic-related stress and consists of 13 items, each on a 5-point Likert scale (1 = strongly disagree, 5 = strongly agree). The higher the summed score, the higher the academic stress. Cronbach’s α was 0.91 in a previous study [36] and 0.86 in this study.

#### 2.3.3. Environmental Factors

Environmental factors included parental support, teacher support, friend support, and bullying victimization. Parental support was measured using the Korean version of the Student Social Support Scale (SSSS) developed by Nolten [37] and adapted by Kim [38]. This scale measures the level of awareness among adolescents about the emotional, informational, evaluative, and material support provided by their parents and consists of 15 items. Parental support was rated from 1 (strongly disagree) to 5 (strongly agree), and the higher the summed score, the higher the level of perceived parental support. Cronbach’s α was 0.97, and the test-retest reliability was 0.75 in a previous study [37]. In this study, Cronbach’s α was 0.95.

Teacher support and friend support were measured using the Social Support Scale-Teacher and the Social Support Scale-Friend developed by Kim [39]. This scale measures the level of awareness among adolescents regarding help, interest, recognition, and encouragement from teachers and friends. Each scale consists of eight items and were rated from 1 (strongly disagree) to 5 (strongly agree). The higher the summed score, the higher the level of perceived support. At the time of development, content validity verification by experts and construct validity verification through factor analysis were performed. Cronbach’s α of the scale was 0.98–0.99, and the test-retest reliability was 0.71–0.78 in a previous study [39]. In this study, Cronbach’s α was 0.87–0.93.

Bullying victimization was measured using the School Victimization Scale developed by Kim [40]. This scale measures the number of victims who experienced bullying and cyberbullying in the past year. For analysis, bullying victimization was categorized into “0 = No, 1 = Yes”. Cronbach’s α was 0.81 in a previous study [40] and 0.77 in this study.

#### 2.3.4. Smartphone Addiction Tendency

Smartphone addiction tendency was measured using the Smartphone Addiction Proneness Scale for Youth (SAPS) [41]. The SAPS consists of four subdomains (difficulty in daily living, virtual life orientation, withdrawal, and tolerance) and 15 items. It was rated on a 4-point Likert scale (1 = strongly disagree, 2 = disagree, 3 = agree, 4 = strongly agree).

Based on the sum of all scores or the sums of subdomain scores, smartphone users were classified into three groups: high risk, latent risk, and normal [4]. Participants were classified as the high-risk group if the sum of all scores was 45 or higher, or their subdomain scores exceeded 16, 13, and 14 for difficulty in daily living, withdrawal, and tolerance, respectively. Participants were classified as the latent risk group if total score was 42–44, or if any of the subdomain scores were satisfied (difficulty in daily living ≥ 14, withdrawal ≥ 12, and tolerance ≥ 13). Other participants were classified as the normal group. Cronbach’s α was 0.88 in a previous study [41] and 0.89 in this study.

In this study, participants in the high risk and latent risk groups were defined as “smartphone addiction tendency group”.

### 2.4. Data Analysis

Data were analyzed using SPSS/WIN 22.0 software (SPSS, Chicago, IL, USA). The variables were analyzed with descriptive statistics. A *t*-test was conducted to investigate the differences in the SAPS between the smartphone addiction tendency group and normal group. The differences in the demographic characteristics, personal factors, and environmental factors according to smartphone addiction tendency were analyzed using χ^2^ test and *t*-test. The correlations among personal factors and environmental factors were analyzed using Pearson’s correlation coefficients. Subsequently, a hierarchical logistic regression analysis was conducted to identify the predictor of smartphone addiction tendency and presented as ORs and 95% confidence intervals (CIs). Model 1 contained demographic characteristics, while personal factors and environmental factors were additionally entered in Models 2 and 3, respectively. The statistical significance level was set at *p* < 0.05.

## 3. Results

### 3.1. Prevalence of Smartphone Addiction Tendency in Adolescents

Of the 502 participants, 17.9% were identified as the tendency group for smartphone addiction and 82.1% were classified as the normal group according to the criteria of the N IA [4]. The tendency group for smartphone addiction showed significantly higher scores on the scale as well as on each subdomain as compared with the normal group (Table 1).

### 3.2. Differences in Demographic Characteristics According to Smartphone Addiction Tendency

Differences in demographic characteristics according to smartphone addiction tendency are as shown in Table 2. Among the demographic characteristics, smartphone addiction showed a statistically significant difference in age (*t* = −2.23, *p* = 0.026), type of school system (*χ^2^* = 8.52, *p* = 0.014), subjective economic level (*χ^2^* = 10.02, *p* = 0.007), and subjective health status (*χ^2^* = 12.97, *p* < 0.001).

### 3.3. Differences in Personal and Environmental Factors According to Smartphone Addiction Tendency

Among the personal and environmental factors, smartphone addiction tendency showed a statistically significant difference in resilience (*t* = 3.95, *p* < 0.001), academic stress (*t* = −4.70, *p* < 0.001), parental support (*t* = 4.25, *p* < 0.001), friend support (*t* = 2.38, *p* = 0.019), and bullying victimization (*χ^2^* = 9.85, *p* = 0.002) (Table 3).

### 3.4. Correlations among Personal Factors, and Environmental Factors

The correlations among independent variables can be found in Table 4. Strong correlations were seen between (a) resilience and parental support, (b) resilience and friend support, and (c) parental support and friend support. The correlation coefficients of all independent variables ranged from −0.31 to 0.48 and confirmed that there was no multicollinearity between the variables since the correlation coefficient was lower than 0.9 [42].

### 3.5. Factors Associated with Smartphone Addiction Tendency

To identify the factors associated with smartphone addiction tendency, a hierarchical logistic regression analysis was performed by including significant variables in the univariate analysis. The results are presented in Table 5.

Model 1 contained significant demographic characteristics: age, type of school system, subjective economic level, and subjective health status. The odds for smartphone addiction tendency increased by approximately 203% with “low” economic level, compared to “high” and “medium”, and increased by 124% with “unhealthy” health status, compared to “healthy”.

The personal factors (resilience, academic stress) were added to Model 2. The odds for smartphone addiction tendency increased by 80% with one unit increase in academic stress.

The environmental factors (parental support, friend support, and bullying victimization) were added to the final model (Model 3). The odds for smartphone addiction tendency increased by approximately 151% with “low” economic level, compared to “high” and “medium”, and increased by 64% with one unit increase in academic stress. The odds for smartphone addiction tendency decreased by 38% with one unit increase in parental support. In addition, the odds for smartphone addiction tendency increased by approximately 134% as the subjects replied “yes” to bullying victimization.

## 4. Discussion

The aim of this study was to identify the related factors of smartphone addiction tendency among adolescents using the ecological model from personal and environmental perspectives. The findings showed that the prevalence of smartphone addiction tendency among Korean adolescents was 17.9% and the factors associated with smartphone addiction tendency were subjective economic level, academic stress, parental support, and bullying victimization.

Among the subjects of this study, the smartphone addiction tendency group including the latent risk and high risk groups accounted for 17.9%, which was different from the 39.6% reported by the NIA [1]. As the differences in the prevalence rate of smartphone addiction tendency derive from different methodologies, subjects, regions, and measuring tools, a follow-up study that has complemented such aspects needs to be conducted. Nevertheless, as prior studies reported that Korean adolescents were more likely to be addicted to smartphone use compared to their overseas counterparts [2,3], it is necessary to carry out systematic research and come up with measures on the causes, prevention, and solutions, considering the social and cultural characteristics of Korean adolescents.

It appeared that in the final model (Model 3), subjective economic status, academic stress, parental support, and bullying victimization were associated with smartphone addiction tendency. 

As a result, smartphone addiction tendency increased with lower subjective economic status. This is in line with the recently announced research result where lower subjective social status led to a higher risk of smartphone addiction [43]. As low subjective socioeconomic status is related to not only psychological issues such as depression, anxiety, and low self-esteem, but also smoking and drinking, adolescents with low subjective social status could be vulnerable to smartphone addiction [43,44]. Lin and Liu [43] reported that adolescents who perceive low subjective social status experience relative privation. This exposes them to the risk of smartphone addiction as they use smartphones to resolve such a deprivative feeling and satisfy basic psychological needs [43]. Also, families with low economic status have difficulty controlling a child’s excessive smartphone use as the parents work for a long time and have less opportunities to participate in after-school classes or leisure activities due to economic issues, which increases adolescents’ time spent on smartphones as they spend more time alone [45,46]. Hence, it is necessary to pay greater attention to adolescents with low objective, or subjective, economic status. However, given that this study measured subjective economic status because adolescents generally did not know the exact income of their families, caution is needed when generalizing the results of the study.

Smartphone addiction tendency of adolescents appeared to grow with greater stress from studying, which is in line with the results of prior studies [16,47]. Adolescents in Korea are exposed to great stress due to factors such as the education system focused on the College Scholastic Ability Test (CSAT), high expectations of parents or teachers, and fierce competition among peers [20]. To relieve stress, they repeatedly use various content provided on smartphones for an extended time, which may lead to smartphone addiction [48]. It was shown that Korean adolescents use their smartphones for 4.8 h per day on average, where 34.2% are spent on recreational activities (games, videos, music, e-books, webcomics, etc.), 32.9% on academic activities, and 25.8% on communication (messengers, Social Media, e-mails) [1,5]. Although smartphone use for recreational activities and communication may temporarily reduce academic stress, it is also associated with low academic accomplishments and smartphone addiction tendency [5,49,50]. As academic stress is inevitable in the educational environment in Korea, it will be the number one task to minimize its negative impact by effectively responding to such stress to prevent smartphone addiction. Based on the results of this study, school health teachers, counselors, and educators need to make interventions (relaxation technique training, psychological counseling, etc.) to help students build greater management skills and resilience against stress.

In the final model, parental support was identified as a protective factor for smartphone addiction tendency. This result is identical to the results of prior studies that reported that support from parents and family is an important protective factor of smartphone or computer game addiction [17,51,52]. Zimmerman and colleagues [53] stated that parents form the most vital system for the health and healthy growth of adolescents, and that their support, communication techniques, and supportive childcare are critical to their psychological development. Furthermore, as recent studies emphasize, parents themselves are an important environmental factor for adolescents, reporting that behaviors such as parental phubbing, adequate supervising, and control affect their smartphone addiction, therefore, parents, social organizations, and communities should make cooperative efforts to create a healthy family environment [54].

In the final model, bullying victimization turned out to be another risk factor for smartphone addiction tendency. This is in line with the results of prior studies that reported that adolescents’ experience of school bullying is a risk factor of smartphone, internet, and game addictions [25,27,55]. The use of smartphones tend to alleviate high levels of stress related to school victimization [25,27]. Also, adolescents are more exposed to being addicted to the internet or smartphone as they immerse in games, online chats, and pornography to resolve internal issues such as depression, anxiety, contraction, intimidation, lethargy, and PTSD (post-traumatic stress disorder) caused by violence [27,55,56]. Adolescents who experienced bullying at school preferred engaging in online interactions where they are anonymous without any pressure, as they find compensation for their psychological desire related to unsatisfied social relationships in the real world, this, in turn, heightens the risk of smartphone addiction [6,48]. In particular, bullying victimization, a type of violence at school, leads to poor psychosocial adaptation, such as poor perception of pro-social peer behaviors, internal problems, and relational aggression [26]. Therefore, it is necessary to make active and regular interventions for students who have been victims of violence in school and to design programs to prevent and educate against violence in school.

In short, adolescents’ smartphone addiction tendency is associated with demographic, personal, and environmental factors as explained in the social ecological model. According to the results of this study, adolescents with low economic status, high academic stress, or low perception of parental support, or those who have been victims of bullying, are more likely to be addicted to smartphones, which requires continued monitoring and management. Therefore, nurses in communities and schools should take a multidimensional approach that assesses and implements all systems surrounding adolescents in a holistic manner in their nursing practice. Also, a preventative program for smartphone addiction of adolescents considering associated factors needs to be developed.

This study is meaningful in that it provides an integrated understanding and theoretical framework for smartphone addiction in adolescents by analyzing associated factors—risk factors and protective factors—for smartphone addiction of adolescents in personal and environmental aspects. This will be the empirical evidence for research on problematic behaviors involving various media dependencies outside the adolescents’ smartphone addiction.

This study has several limitations. First, as this is a cross-sectional study, the cause-and-effect relationship among major variables could not be identified. Hence, future research needs to establish a longitudinal path model including the causal relationship between risk and protective factors and identify the relationship. Second, the samples were obtained by convenience sampling methods; thus, the study results have limited generalizability and should be interpreted with caution. Lastly, as the study looked at the environmental factors at a personal level to figure out how such factors affect smartphone addiction of adolescents, the relationship between unique characteristics of schools or local communities and smartphone addiction could not be identified. As the multilevel model is adequate for the identification of environmental factors’ contextual effect at a macroscopic level [57], it is necessary to look at how environmental factors at a personal or group level affects adolescents’ smartphone addiction based on big data.

## 5. Conclusions

To suggest empirical evidence for the effective prevention and management of adolescents’ smartphone addiction, this study examined factors relevant to the smartphone addiction tendency of Korean adolescents.

As a result, smartphone addiction tendency among Korean adolescents was found to be associated with subjective economic status, academic stress, parental support, and bullying victimization. Based on the results of this study, a program that considers multidimensional aspects of adolescents such as effective management of academic stress, prevention of bullying, and increased parental support, should be developed and adopted to prevent smartphone addiction in adolescents.

It is important to encourage adolescents to build the capacity to successfully respond to stressful situations. This should be backed by parental support as well as a supportive environment of schools and communities.

## Figures and Tables

**Table 1:** Prevalence of smartphone addiction in adolescents.

	SA Tendency Group	Normal Group	Total	*t*
	(*n* = 90)	(*n* = 412)	(*n* = 502)	
	*n* (%) or Mean ± SD	*n* (%) or Mean ± SD	*n* (%) or Mean ± SD	
SA tendency group	90 (17.9)	412 (82.1)		
High risk group	37 (7.4)			
Latent risk group	53 (10.5)			
**Smartphone addiction proneness scale score**				
Total scores	41.33 ± 5.68	29.06 ± 6.20	31.26 ± 7.72	18.24 ***
Difficulty in daily living	14.37 ± 3.08	10.51 ± 2.66	11.21 ± 3.11	12.04 ***
Virtual life orientation	4.64 ± 1.23	3.11 ± 0.98	3.38 ± 1.18	11.02 ***
Withdrawal	11.62 ± 2.21	7.64 ± 2.02	8.35 ± 2.56	16.59 ***
Tolerance	11.92 ± 2.28	8.68 ± 2.23	9.26 ± 2.56	12.39 ***

SA = smartphone addiction; *** *p* < 0.001.

**Table 2:** Comparison of demographic characteristics between the smartphone addiction tendency group and normal group.

Variables	Categories	Total	SA Tendency Group	Normal Group	*χ*^2^ or *t*
		(*n* = 502)	(*n* = 90)	(*n* = 412)	
		*n* (%) or	*n* (%) or	*n* (%) or	
		Mean ± SD	Mean ± SD	Mean ± SD	
Sex	Male	227 (45.2)	34 (37.8)	193 (46.8)	2.45
	Female	275 (54.8)	56 (62.2)	219 (53.2)	
Age (year)		13.00 ± 0.80	13.17 ± 0.89	12.96 ± 0.77	−2.23 *
Type of school system	Co-education school	303 (60.4)	65 (72.2)	238 (57.8)	8.52 *
	Boys’ school	78 (15.5)	6 (6.7)	72 (17.5)	
	Girls’ school	121 (24.1)	19 (21.1)	102 (24.8)	
Structure of family	Intact family	422 (84.1)	72 (80.0)	350 (85.0)	1.35
	Single parent	80 (15.9)	18 (20.0)	62 (15.0)	
Parents working status	Both parents working	303 (60.4)	55 (61.1)	248 (60.2)	0.03
	Single parent working/Other	199 (39.6)	35 (38.9)	164 (39.8)	
Subjective economic level	Low	29 (5.8)	11 (12.2)	18 (4.4)	10.02 **
	Medium	349 (69.5)	63 (70.0)	286 (69.4)	
	High	124 (24.7)	16 (17.8)	108 (26.2)	
Subjective health status	Unhealthy	73 (14.5)	24 (26.7)	49 (11.9)	12.97 ***
	Healthy	429 (85.5)	66 (73.3)	363 (88.1)	

SA = Smartphone addiction; * *p* < 0.05; ** *p* < 0.01; *** *p* < 0.001.

**Table 3:** Difference in study variables based on smartphone addiction tendency.

Variables	Categories	Total	SA Tendency Group	Normal Group	*χ*^2^ or *t*
		(*n* = 502)	(*n* = 90)	(*n* = 412)	
		*n* (%) or Mean ± SD	*n* (%) or Mean ± SD	*n* (%) or Mean ± SD	
**Personal factors**					
Resilience		4.89 ± 1.01	4.52 ± 1.08	4.98 ± 0.97	3.95 ***
Academic stress		2.44 ± 0.76	2.78 ± 0.80	2.37 ± 0.73	−4.70 ***
**Environmental factors**					
Parental support		3.93 ± 0.76	3.58 ± 0.91	4.01 ± 0.70	4.25 ***
Teacher support		3.65 ± 0.70	3.58 ± 0.82	3.66 ± 0.67	0.85
Friend support		3.91 ± 0.74	3.72 ± 0.89	3.95 ± 0.70	2.38 *
Bullying victimization	No	460 (91.6)	75 (83.3)	385 (93.4)	9.85 **
	Yes	42 (8.4)	15 (16.7)	27 (6.6)	

SA = Smartphone addiction; * *p* < 0.05; ** *p* < 0.01; *** *p* < 0.001.

**Table 4:** Correlations among personal factors and environmental factors.

	Resilience	Academic Stress	Parental Support	Teacher Support	Friend Support
Resilience	1				
Academic stress	−0.31 ***	1			
Parental support	0.48 ***	−0.31 ***	1		
Teacher support	0.32 ***	−0.23 ***	0.33 ***	1	
Friend support	0.45 ***	−0.19 ***	0.41 ***	0.29 ***	1

*** *p* < 0.001.

**Table 5:** Factors associated with smartphone addiction tendency.

	Model 1		Model 2		Model 3	
	OR	95% CI	OR	95% CI	OR	95% CI
Age (year)	1.233	0.917–1.658	1.116	0.820–1.519	1.148	0.839–1.569
Type of school system	1.626	0.962–2.748	1.785	1.041–3.062 *	1.592	0.914–2.773
(Co-education, ref. = boys’, girls’)						
Subjective economic level	3.059	1.336–7.004 **	2.680	1.125−6.383 *	2.514	1.038−6.090 *
(low, ref. = medium, high)						
Subjective health status	2.241	1.264−3.976 **	1.805	0.992−3.283 *	1.801	0.985−3.295
(unhealthy, ref. = healthy)						
Resilience			0.780	0.605−1.004	0.852	0.639−1.135
Academic stress			1.840	1.302−2.599 **	1.636	1.147−2.335 **
Parental support					0.622	0.431−0.899 *
Friend support					1.094	0.750−1.596
Bullying victimization					2.347	1.101−5.004 *
(yes, ref. = no)						
*χ^2^* (*p*)	24.564 (<0.001)		45.981 (<0.001)		57.202 (<0.001)	
Hosmer & Lemeshow *χ^2^* (*p*)	1.498 (0.960)		9.376 (0.312)		6.967 (0.540)	
Nagelkerke’s *R*^2^	0.078		0.144		0.198	

* *p* < 0.05; ** *p* < 0.01.

## Data Availability

The data presented in this study are available on request from the author. The data are not publicly available due to the protection of the privacy of research subjects.
